# Inhibition of nucleo-cytoplasmic proteasome translocation by the aromatic amino acids or silencing Sestrin3—their sensing mediator—is tumor suppressive

**DOI:** 10.1038/s41418-024-01370-x

**Published:** 2024-09-12

**Authors:** Ido Livneh, Bertrand Fabre, Gilad Goldhirsh, Chen Lulu, Adar Zinger, Yael Shammai Vainer, Maya Kaduri, Aviva Dahan, Tamar Ziv, Avi Schroeder, Yinon Ben-Neriah, Yaniv Zohar, Victoria Cohen-Kaplan, Aaron Ciechanover

**Affiliations:** 1 The Rappaport Technion Integrated Cancer Center (R-TICC) and the Rappaport Faculty of Medicine and Research Institute, Technion-Israel Institute of Technology, Haifa, Israel; 2 Institute of Pathology and Cytology, Rambam Health Care Campus, Haifa, Israel; 3 The Lautenberg Center for Immunology and Cancer Research, Institute of Medical Research Israel-Canada, Hebrew University-Hadassah Medical School, Jerusalem, Israel; 4 The Louis Family Laboratory for Targeted Drug Delivery and Personalized Medicine Technologies, Faculty of Chemical Engineering, Technion-Israel Institute of Technology, Haifa, Israel; 5 Smoler Proteomic Center, Faculty of Biology, Technion-Israel Institute of Technology, Haifa, Israel; 6 Present Address: Laboratoire de Recherche en Sciences Végétales, UMR5546, Université de Toulouse 3, INP, CNRS, Auzeville-Tolosane, France

**Keywords:** Cancer, Biochemistry

## Abstract

The proteasome, the catalytic arm of the ubiquitin system, is regulated via its dynamic compartmentation between the nucleus and the cytoplasm, among other mechanisms. Under amino acid shortage, the proteolytic complex is translocated to the cytoplasm, where it stimulates proteolysis to supplement recycled amino acids for essential protein synthesis. This response is mediated via the mTOR pathway and the lack of the three aromatic amino acids Tyr, Trp, and Phe (YWF). mTOR activation by supplementation of the triad inhibits proteasome translocation, leading to cell death. We now show that tumoral inherent stress conditions result in translocation of the proteasome from the nucleus to the cytosol. We further show that the modulation of the signaling cascade governed by YWF is applicable also to non-starved cells by using higher concentration of the triad to achieve a surplus relative to all other amino acids. Based on these two phenomena, we found that the modulation of stress signals via the administration of YWF leads to nuclear proteasome sequestration and inhibition of growth of xenograft, spontaneous, and metastatic mouse tumor models. In correlation with the observed effect of YWF on tumors, we found – using transcriptomic and proteomic analyses – that the triad affects various cellular processes related to cell proliferation, migration, and death. In addition, Sestrin3—a mediator of YWF sensing upstream of mTOR—is essential for proteasome translocation, and therefore plays a pro-tumorigenic role, positioning it as a potential oncogene. This newly identified approach for hijacking the cellular “satiety center” carries therefore potential therapeutic implications for cancer.

## Introduction

Degradation of a protein by the ubiquitin-proteasome system (UPS) is carried out in two sequential steps: (i) recognition of the substrate and its covalent marking by ubiquitin, mediated by the ubiquitin-activating enzyme (E1), a ubiquitin-conjugating enzyme (E2), and a specific ubiquitin ligase (E3) [1, 2]; and (ii) binding and degradation of the tagged protein by the 26S proteasome with release of reusable ubiquitin [3, 4]. Regulation of the different stages of protein degradation by the UPS was extensively studied. Examples include substrate recognition by their cognate E3 ligases [5, 6], the conjugation of ubiquitin moieties as well as the length and nature of the ubiquitin chains [7, 8], and the removal of ubiquitin by deubiquitinating enzymes (DUBs) [9, 10]. Regulatory mechanisms of the proteasome, such as its assembly [11, 12], composition [13, 14], recognition of ubiquitinated substrates [15–17], and post-translational modifications [18–21] were also studied. Recent studies showed that proteasome function alters in response to different environmental conditions, including metabolic stress and nutrient availability [22–25].

The mechanistic target of rapamycin (mTOR) is a sensor for various environmental cues, acting as an integrating hub for different input signals, relaying the appropriate output signals via the different downstream branches of its cascade [26]. While mTOR is activated under favorable environmental conditions such as nutrient and oxygen availability [27, 28], it is inhibited to varying extents under shortage. Specifically, inhibition of mTOR due to metabolite deprivation was shown to upregulate catabolism [29], including protein degradation via both the UPS and autophagy [30, 31].

We have recently described an as yet unknown regulatory mechanism of the UPS [32], where the proteasome is translocated from the nucleus to the cytosol in response to amino acid starvation, a process regulated via the mTOR signaling pathway. This stress-coping mechanism is governed by as yet undescribed mTOR-agonistic amino acids – Tyr, Trp, and Phe (Y, W, and F). The sensing of the triad is mediated by Sestrin3 (SESN3), an interactor of the GATOR2 complex, which acts upstream of mTOR [33]. Interestingly, the previously known agonistic amino acids of mTOR, namely Gln, Leu, and Arg [34–37] (QLR), did not have an effect on proteasome translocation. In line with these findings, also Sestrin2, a receptor of Leu [38] does not to play a role in the mTOR signaling cascade that governs proteasome dynamics [32]. We showed in cultured cancer cell lines that proteasome translocation to the cytosol stimulates protein degradation, supplying starved cells with recycled amino acids, which are required for the synthesis of essential proteins [39]. Notably, activation of mTOR with only YWF was sufficient to inhibit proteasome recruitment, leading to its sequestration in the nucleus. This, in turn, deprived cells from amino acids otherwise generated by the proteasome, and resulted in cell death [32].

We now report that proteasome recruitment as a stress-coping mechanism is occurring also in vivo, in various animal tumor models, and that YWF administration induces apoptosis and tumor shrinkage. Further, we found that SESN3 is essential for this process, as its deletion inhibits proteasome translocation resulting in arresting tumor growth. This positions SESN3 as a potential oncogene and possible therapeutic target. Mechanistically, transcriptomic and proteomic analyses of the YWF signal show that it has broad effects on various cellular functions determining cell fate.

## Results

### Proteasome recruitment is specific, yet is not limited to nutritional stress

**Fig. 1:**
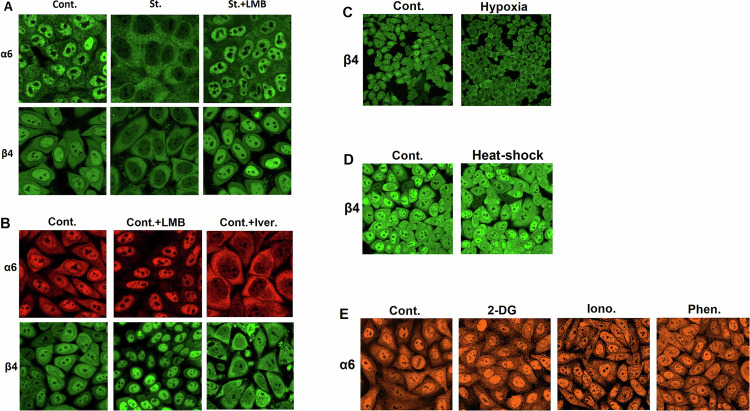
Stress-induced translocation of the proteasome from the nucleus to the cytosol is specific but not limited to amino acid shortage. **A** HeLa cells were cultured in a complete medium (Cont.) or a starvation medium lacking amino acids in the absence (St.) or presence of Leptomycin B (St.+LMB). The indicated proteasome subunits were visualized via confocal microscopy. **B** HeLa cells were cultured in a complete medium alone (Cont.), or in the presence of LMB (Cont.+LMB) or ivermectin (Cont.+Iver.), and the proteasome was visualized as above. **C** HeLa cells were cultured in a complete medium in either 21% (Cont.) or <1% O_2_ (Hypoxia), and the proteasome was visualized as above. **D** Hela cells were subjected to heat-shock, and the proteasome was visualized as above. **E** HeLa cells were treated with either 2-deoxyglucose (2-DG), ionomycin (Iono.), or phenformin (Phen.), and the proteasome was visualized as above.

We have shown recently that the proteasome is actively shuttled between the nucleus and the cytoplasm under steady state, probably fine-tuning proteolysis in the ever-changing metabolic and environmental conditions. We further found that following amino acid starvation the proteasome is translocated from its large nuclear pool to the cytoplasm (Figs. 1A, B and S1A, B), where it facilitates protein breakdown and provision of amino acids [32]. Next, it was important to test whether proteasome recruitment in response to starvation is stimulus-specific, especially in light of the various stress conditions experienced by tumors. For example, poorly perfused tumor cells are also subjected to low oxygenation which constitutes a challenge to the tumor, and thereby a potential weakness that may be used as a therapeutic target [40]. The UPS is already known to play a role in cellular adjustment to altered levels of oxygen, as in the case of proteasomal degradation of the Hypoxia-Inducible Factor 1α (HIF1α), which is removed under normoxia while being stabilized under hypoxia [41]. Interestingly, it was also suggested that hypoxia induces broader alterations in the function of the UPS [42]. In light of the relationship between proteasomal activity and oxygen levels, we hypothesized that hypoxia may lead to proteasome translocation to the cytoplasm. Indeed, we found that this is the case (Figs. 1C and S1C). In contrast, stress conditions such as heat shock (Fig. 1D and S1D) and the activation of catabolic signals *via* AMPK (Figs. 1E and S1E) did not result in proteasome export. Interestingly, these observations further distinguish starvation-induced translocation in mammalian cells from the formation of proteasome storage granules in yeast following glucose starvation – which is mediated *via* AMPK [43]. Taken together, nucleo-cytoplasmic proteasome shuttling seems specific, and most probably serves a pathophysiological role (see below).

### The YWF signal affects various cellular pathways

**Fig. 2:**
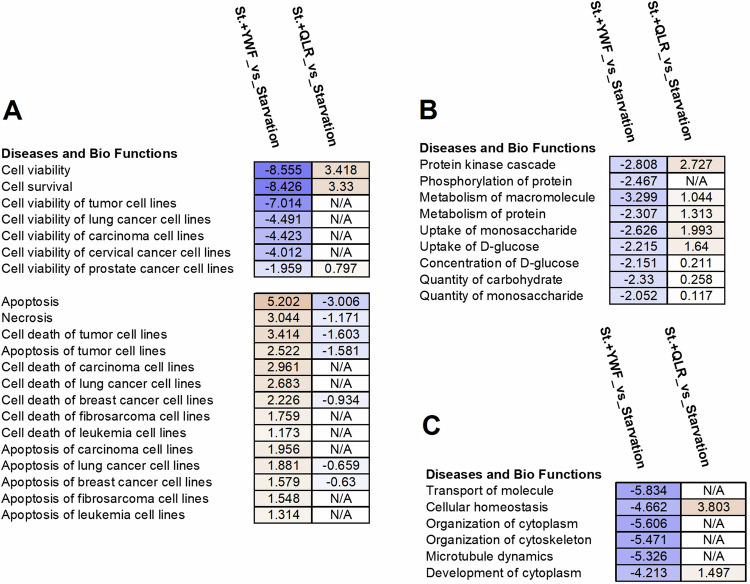
The effect of YWF on gene expression. HeLa cells were incubated in either a complete medium, starvation medium, starvation medium supplemented with Tyr, Trp, and Phe (St.+YWF), or starvation medium supplemented with Gln, Leu, and Arg (St.+QLR). Cells were harvested, RNA was extracted and sequenced, and differentially expressed genes were compared according to the Ingenuity Pathway Analysis (see “Materials and methods” for further details). The altered functions are clustered according to cell viability/death (**A**), metabolism (**B**), and general cell organization and homeostasis (**C**). Presented are biological functions that were found to be affected differentially in response to the addition of YWF (St.+YWF_vs_Starvation) or QLR (St.+QLR_vs_Starvation).

In light of the cytotoxic effect of YWF on stressed cancer cells, we next aimed to unravel the biological functions which are specifically affected by the aromatic triad. To that end, we used RNA-Seq. and analyzed transcriptomes of cells which were incubated in either a complete medium (Control), a medium lacking all amino acids (Starvation), or a medium lacking all amino acids except for YWF (Starvation+YWF) or QLR (Starvation+QLR). The latter were used in order to identify differentially expression genes (DEGs) which are unique to the effect of the aromatic YWF. We found that YWF downregulated the expression of transcripts associated with cell survival and viability, and upregulated those associated with functions such as apoptosis and necrosis (Fig. 2A). Importantly, none of these effects were observed following the addition of QLR, partially explaining our previous report that while YWF induce extensive cell death, QLR do not [32]. While evaluating functions other than survival/apoptosis, we found that YWF downregulated kinase-associated signaling cascades and phosphorylation, metabolism of macromolecules, proteins, and different carbohydrates (Fig. 2B), as well as other homeostatic processes (Fig. 2C) and pro-tumorigenic activities (Fig. S2A). The direct contribution of these functions to YWF-regulated cell fate is yet to be explored. In any case, the general trend of transcriptomic alterations induced by YWF is in line with our report that YWF create a false satiety signal via mTOR, preventing cancer cells from recruiting essential coping mechanisms. All-in-all, the transcriptomic analysis revealed that the cellular effect of the signal governed by YWF is much broader than pathways related solely to amino acids metabolism (Fig. S2B).

**Fig. 3:**
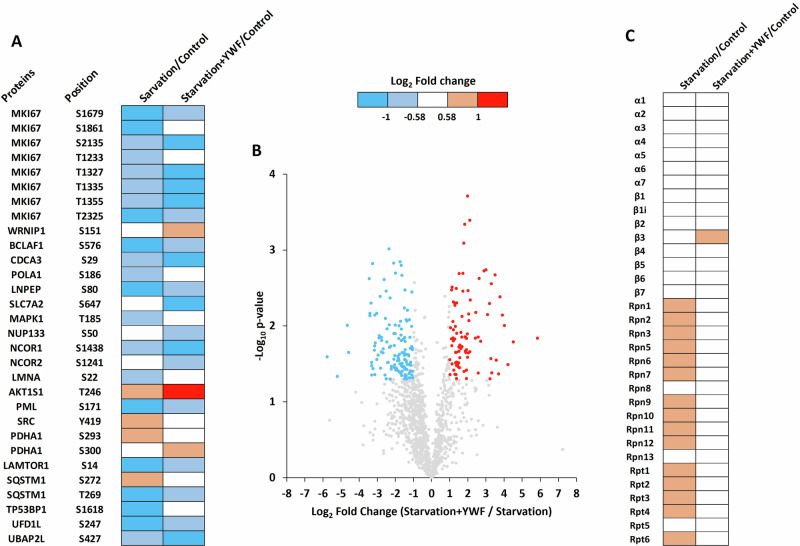
YWF supplementation modulates post-translational modifications and proteasome assembly. Proteomic experiments were performed to assess changes in protein phosphorylation (**A**), ubiquitination (**B**), and proteasome composition (**C**), following YWF supplementation. Three biological replicates were performed for each experiment, and a two-tailed Welch’s *t*-test was used. **A** Differentially phosphorylated proteins are presented in a heat map displaying the Log_2_ fold-change (*Starvation/Control*, and *Starvation* + *YWF/Control*) for each phosphorylation site. Gene names and phosphorylated residues are presented. **B** Differentially ubiquitinated proteins are presented in a volcano plot displaying the log_2_ fold-change (*Starvation* + *YWF/Starvation*) for each ubiquitination site quantified, and the corresponding −Log_10_ p-value. Blue dots represent ubiquitination sites more abundant in starved cells in the absence of YWF; red dots represent those more abundant in the presence of YWF; gray dots represent sites that are not differentially ubiquitinated. **C** Cell lysates were subjected to immunoprecipitation of the 20S proteasome using an antibody for the α6 subunit. The differentially interacting proteasomal subunits are presented in a heat-map displaying the Log_2_ fold-change (*Starvation/Control*, and *Starvation* + *YWF/Control*) for each proteasome subunit. The blue, red, and white colors represent a decrease, increase, or no change in the interaction with the α6 subunit, respectively.

In light of the above findings concerning the transcriptomic effects of YWF, and the previous findings concerning the role of the UPS in shaping the proteome under stress [44], we aimed to explore the consequences of YWF supplementation at the protein level. Using mass spectrometry (MS)-based proteomics, we have previously shown that YWF-induced proteasome nuclear sequestration, stabilized nearly 1,000 proteins, the level of which decreased under starvation. Most of these proteins were found to be cytosolic [32]. We now aimed to broaden this observation to alterations in the UPS and other intracellular signaling pathways. To that end, the levels of protein ubiquitination and phosphorylation were monitored following starvation in either the presence or absence of YWF. First, we monitored changes in protein phosphorylation following treatment with YWF, identifying thirty phosphosites on 22 proteins that were differentially phosphorylated in the presence of the triad (Fig. 3A). These proteins are involved in apoptosis (TP53BP1 and BCLAF1), cell cycle (MKI67 and CDCA3), and intracellular signaling pathways, including the mTOR cascade (MAPK1, AKT1S1, LAMTOR1, and SRC; Fig. 3A). Next, we assessed significant changes in protein ubiquitination in response to YWF (Fig. 3B). For instance, increased ubiquitination was observed on several proteins involved in transport and cytoskeletal organization (Fig. S3), which is in correlation with the reduced expression of components of similar function, observed in our transcriptomic analyses (Fig. 2D). Conversely, decreased ubiquitination in the presence of YWF was found for proteins involved in proteolysis (proteasome subunits, E3 ubiquitin ligases, and deubiquitinases), chromosome organization, cellular response to DNA damage, and amino acid transport (SLC7A5/LAT1 and SLC3A2/CD98hc, which are also known to dimerize; Fig. S4). Finally, 20S proteasome immunoprecipitation and MS analysis were performed in order to study the effect of YWF on the composition of proteasome complexes. We observed an increase in the association between the 19S regulatory particle and the 20S core particle of the proteasome following starvation (Fig. 3C), which is in line with increased proteasomal activity under shortage [32]. This increase in proteasome assembly was reversed by the addition of YWF (Fig. 3C). This further demonstrates the ability of the triad to “hijack” the stress signaling that would otherwise upregulate proteasome activity in the cytoplasm. As expected, the co-immunoprecipitation of 20S subunits with a6 was virtually unchanged, demonstrating that the 20S core particle by itself is stable (Fig. 3C). Overall, our MS analyses point out to significant modulation of the landscape of both ubiquitome and phospho-proteome in response to YWF supplementation.

**Fig. 4:**
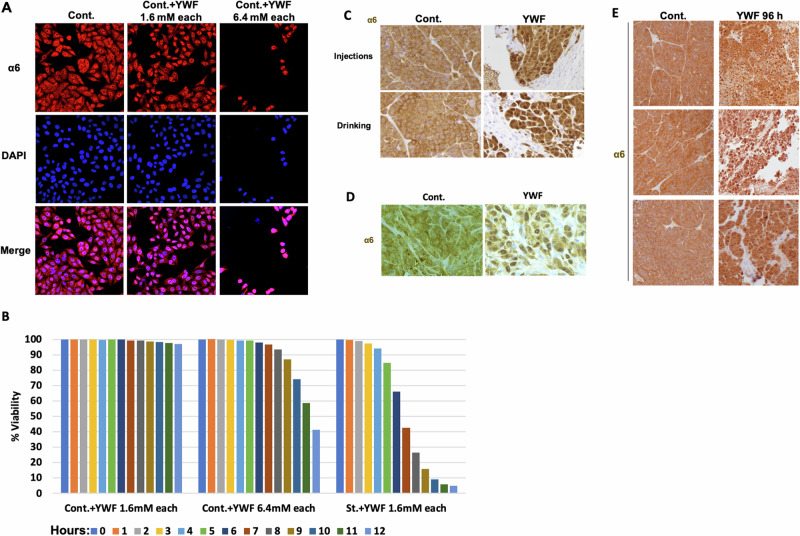
The YWF-stimulated cascade governs proteasome dynamics also in non-starved cells and animal tumor models. **A** HeLa cells were cultured in a complete medium (Cont.), or in the presence of the indicated concentrations of YWF. The proteasome was visualized using confocal microscopy. **B** HeLa cells were incubated in different YWF concentrations added to a complete DMEM medium in order to assess the cytotoxic effect of the aromatic triad on non-starved cells. Cell viability was quantified using high-throughput fluorescent microscopy. HeLa (**C**, **E**) and MDA-MB-231 (**D**) cells were inoculated in immune-compromised mice to generate xenograft tumors, and animals in the treatment group were administered with YWF (see Materials and methods:lly for the proteasome. *Injections* refer to the administration of treatment subcutaneously to the tumor bed, while *Drinking* refers to its administration orally, via the animals’ drinking water.

Since YWF was found to regulate key cellular functions, some of which are not directly related to amino acid starvation, we next tested their effect on proteasome subcellular distribution in non-starved cells. We found that the addition of excess YWF to well-fed cells resulted in further accumulation of the proteasome in the nucleus in a dose-dependent manner. Interestingly, the YWF concentration which was sufficient to induce a near-complete cell death when added to a starvation medium (otherwise containing no Y, W, or F), had only a partial cytotoxic effect when added to the complete medium (where the aromatic, and all other amino acids are already present; Fig. 4A, B). Nevertheless, creating a significant excess of YWF on top of the preexisting concentration of the triad in the complete medium, resulted in both nuclear proteasome sequestration and cell death (Fig. 4A, B).

### Proteasome recruitment is essential for tumor growth, and its inhibition results in cell death and reduction in tumor size

After finding that proteasome translocation is induced also by stress conditions other than starvation (Fig. 1C), that YWF regulate various key functions (Fig. 2), and that the triad can induce proteasome accumulation in the nucleus and cell death also in non-starved cells (Fig. 4A, B), we hypothesized that proteasome recruitment to the cytosol—and its inhibition by YWF—may occur also in vivo, in the intact animal. Notably, solid tumors are characterized by poor perfusion and oxygenation, and cancer cells are inherently stressed [45]. We postulated that this stress may lead to proteasome recruitment to the cytoplasm, and that the metabolic requirements of rapidly dividing tumor cells will render them more vulnerable to forced nuclear sequestration of the proteasome by YWF. To test this hypothesis, we used human breast and uterine cervix tumor models in mice, finding that indeed, the stressed tumor cells exhibit cytosolic localization of the proteasome (Fig. 4C–E). Intriguingly, we found that treating the animals with YWF, either via injection to the tumor bed or through their drinking water, led to nuclear accumulation of the proteasome, similar to the effect observed in cultured cells (Fig. 4C–E). The effect of YWF was seen already following 96 h of treatment (Fig. 4E), suggesting that proteasome distribution is dynamic also in vivo. Consequent to YWF-induced nuclear proteasome sequestration, we observed large areas of tumor tissue destruction in the xenografts, and the replacement of tumor cells by mouse cells that are not stained by the antibody used (Fig. 4C; see also below).

**Fig. 5:**
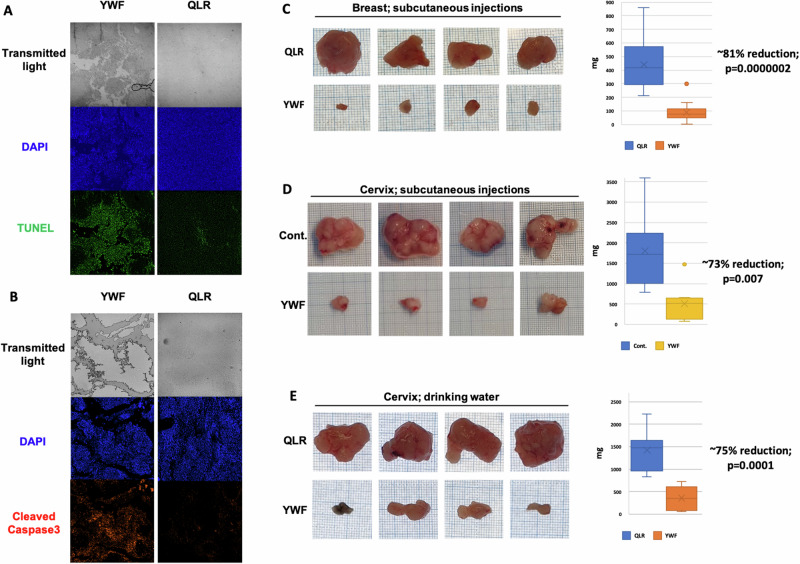
YWF-induced proteasome nuclear sequestration results in cell death and tumor shrinkage. **A** HeLa cells were inoculated in immune-compromised mice to generate xenograft tumors, and animals were treated as indicated (see also under “Materials and methods”). Tumors were harvested and tissue sections were fixed in formaldehyde and embedded in paraffin. Presented is the detection of apoptosis using TUNEL staining of histopathological slides. **B** Histopathological slides were generated as in A, and presented is the detection of apoptosis using staining for cleaved Caspase3. Xenograft tumors were generated as above, using either MDA-MB-231 (**C**) or HeLa (**D**) cells. Animals were treated as indicated via subcutaneous injections to the tumor bed. Harvested tumors were weighed and photographed for scale on a graph paper. Plotted are tumor weights at the time of mouse sacrificing. **E** Xenograft tumors originating from HeLa cells were generated as in (**D**), and the indicated amino acids were administered via drinking water. Analyses were carried out as in (**D**).

To shed light on the destructive process, we stained tumors for the apoptotic markers TUNEL and cleaved-Caspase3. We found that concomitantly with their induction of proteasome nuclear accumulation, YWF exerted also a wide cytotoxic effect on tumor cells, and that areas stained positive for the apoptotic markers also demonstrated an architecture typical to damaged tissue (Fig. 5A, B). Observing the tumors macroscopically and comparing their weight, we found that the effect of YWF at the cellular level (i.e., proteasome nuclear retainment and apoptosis) is accompanied also by a significant reduction of up to ~80% in tumor size, compared to control tumors (Fig. 5C–E). YWF inhibited efficiently tumor growth regardless of their route of administration (subcutaneously injected or dissolved in drinking water). YWF were effective even when administrated late in the course of tumor development, in which case tumors were allowed to reach a significantly large size prior to the initiation of treatment (Fig. S5A–C).

**Fig. 6:**
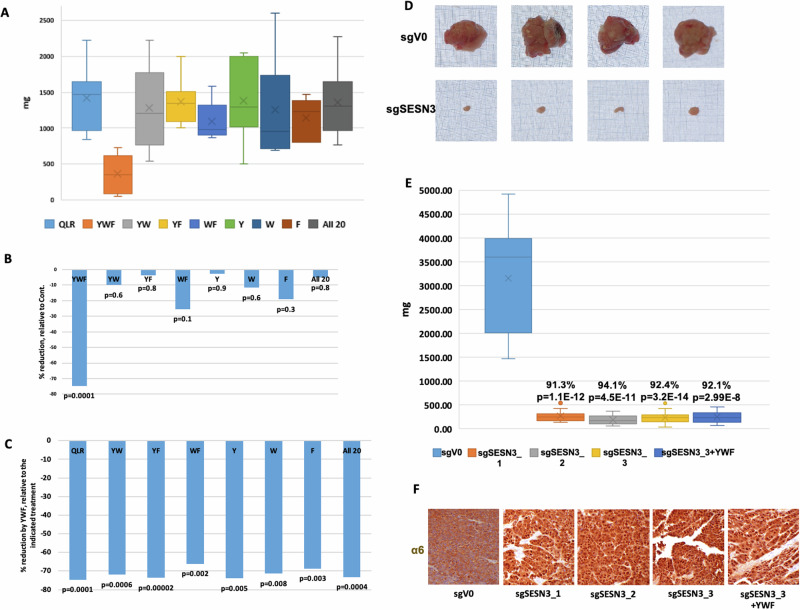
SESN3, mediator of YWF sensing, is a pro-tumorigenic protein. **A**–**C**. HeLa cells were inoculated in immune-compromised mice to generate xenograft tumors, and animals were treated as indicated. Tumors were harvested and tissue sections were fixed in formaldehyde and embedded in paraffin. Plotted are tumor weights at the time of mouse sacrificing (**A**), the reduction in weight following each treatment relative to Control (**B**), and the reduction in weight by YWF, relative to each of the other indicated combinations of amino acids (**C**). **D**–**F** Cells underwent gene editing using CRISPR to generate several clones with knockout of SESN3 (see Livneh et al. [32]). The different clones were inoculated in immune-compromised mice to generate xenograft tumors, and animals were treated as indicated (see also under “Materials and methods”). Tumors were harvested and photographed for scale on a graph paper (**D**). Presented are tumor weights at the time of mouse sacrificing (**E**), and immunohistochemical staining of the proteasome in tumor sections (**F**).

In order to test whether all three aromatic amino acids are essential for the efficient anti-tumoral effect, we next treated mice through their drinking water with all combinations of Tyr, Trp, and Phe—individual amino acids as well as all possible pairs. As in cultured cells [32], we found that only the three of them together induced a significant reduction in tumor size (Fig. 6A, B), and that the trio was far superior to any other combination (Fig. 6C). Importantly, administration of all twenty amino acids had no effect on tumor growth (Fig. 6A, B), underscoring that an excess of the trio relative to the other amino acids is key for the anti-tumoral effect.

### Proteasome sequestration identifies Sestrin3 as a pro-tumorigenic protein

We next tested the possible role of SESN3, which we have recently identified as a mediator of the YWF signal [32], also in vivo. Since in cultured cells lacking SESN3, the proteasome was not recruited to the cytosol under stress [32], one may postulate that tumors in which SESN3 is knocked out will fail to withstand the metabolic stress experienced by cancer cells. We therefore used 3 independent clones of SESN3-KO HeLa cells, which were implanted as xenografts. We found that tumors lacking SESN3 are dramatically smaller, and as expected – the proteasome is localized mostly to their cell nuclei without any treatment (Fig. 6D–F). Since the ability of cells to cope with stress by recruiting the proteasome to the cytosol is abrogated in the absence of SESN3, treating such tumors with YWF had no additive effect, compared to that seen by SESN3 KO (Fig. 6F).

### Sequestering the proteasome in the nucleus is an effective anti-tumoral treatment also in endogenous and metastatic neoplasms in mice

**Fig. 7:**
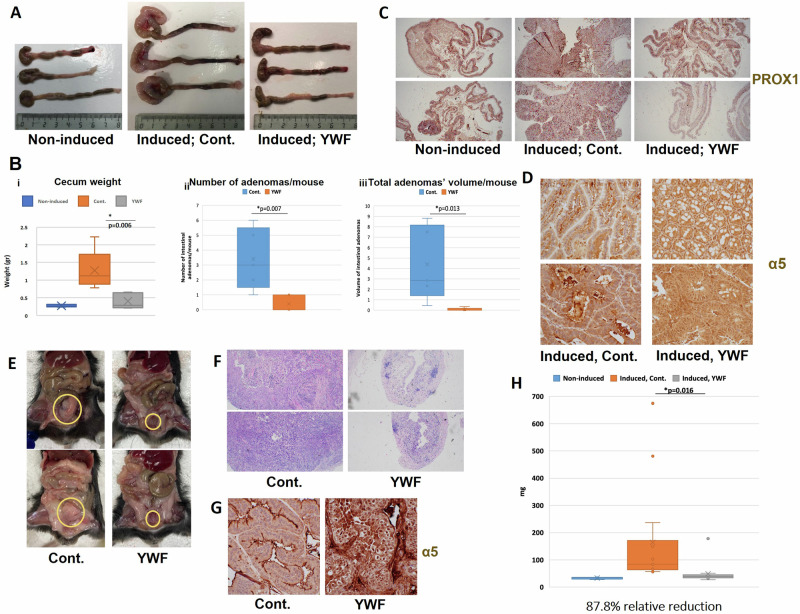
Preventing proteasome recruitment inhibits endogenous tumor growth and metastasis. **A** Gastrointestinal tumors were induced in immune-competent mice via the loss of the tumor suppressor gene adenomatous polyposis coli (APC; see under “Materials and methods”). Colons and cecums from either non-induced mice, mice in which the loss of the tumor suppressor APC was induced and were either untreated (Cont.) or treated with YWF dissolved in their drinking water. **B** Tumors generated and treated as in A were harvested, and tissue sections were fixed in formaldehyde and embedded in paraffin. Presented are the plotting of average cecum weight (i), number of tumors along the colon (ii), and their total volume (iii) at the time of mouse sacrificing. **C** Low magnification of cecums from mice from the indicated groups stained for the high-grade dysplasia marker PROX1. **D** Immunohistochemistry of the proteasome in histopathological sections from the tumors described under **A,** **B**. **E** The carcinogen BBN was administered to immune-competent mice via their drinking water. Following the formation of urine bladder neoplasms, animals in the treatment group were administered with YWF (see also under “Materials and methods”). Mice were sacrificed, bladders were harvested and tissue sections were fixed in formaldehyde and embedded in paraffin. Presented are gross samples at the time of sacrifice. **F** Low magnification of H&E staining of bladders from the different experimental groups. **G** Immunohistochemistry of the proteasome in histopathological section from **E**. **H** Plotting of weights of the bladders harvested in **E**.

Although xenografts serve as a well-established cancer model in animals, they have some inherent limitations. While providing information about tumors from human origin, their vascular and stromal structures do not mimic that of endogenous tumors. We therefore tested our findings also in endogenous tumor models in mice. First, we used a model for colorectal cancer (CRC) induced by loss of the Adenomatous Polyposis Coli (APC) tumor suppressor, as can be found in most cases of CRC in humans [46]. While inducing APC loss resulted in tumors both in the cecum and along the colon of mice, YWF administration reduced the size of the main cecal mass, as well as the number and size of the smaller neoplasms along the colon (Fig. 7A, B). Staining for PROX1, a marker for high grade dysplasia, demonstrated that YWF reduces the extent of neoplastic tissue in the intestine (Fig. 7C). Staining for the proteasome validated, as expected, that YWF treatment resulted in proteasome seclusion in the nucleus (Fig. 7D). Importantly, no damage to normal tissues was observed, including in the liver and kidneys (Fig. S6), underscoring the selectivity of YWF towards cancerous cells.

In another model of an epithelial solid tumor, we used the carcinogen N-Butyl-N-(4-hydroxybutyl)nitrosamine (BBN), which induces bladder carcinoma following prolonged administration via drinking water [47]. After 14 weeks of continuous administration of the carcinogen, a time by which carcinoma in situ and early invasion are present [47], we initiated treatment with YWF. After a total of 25 weeks, the mice were sacrificed, and their bladders were examined. While significant tumors developed as a result of the BBN treatment, monitoring the bladders macroscopically and under a small magnification, showed that the YWF treatment resulted in bladders of nearly normal size (Fig. 7E and F). Staining for the proteasome showed its nuclear sequestration following treatment (Fig. 7G), and weighing at the different bladders demonstrated a significant reduction in tumor mass (Fig. 7H).

**Fig. 8:**
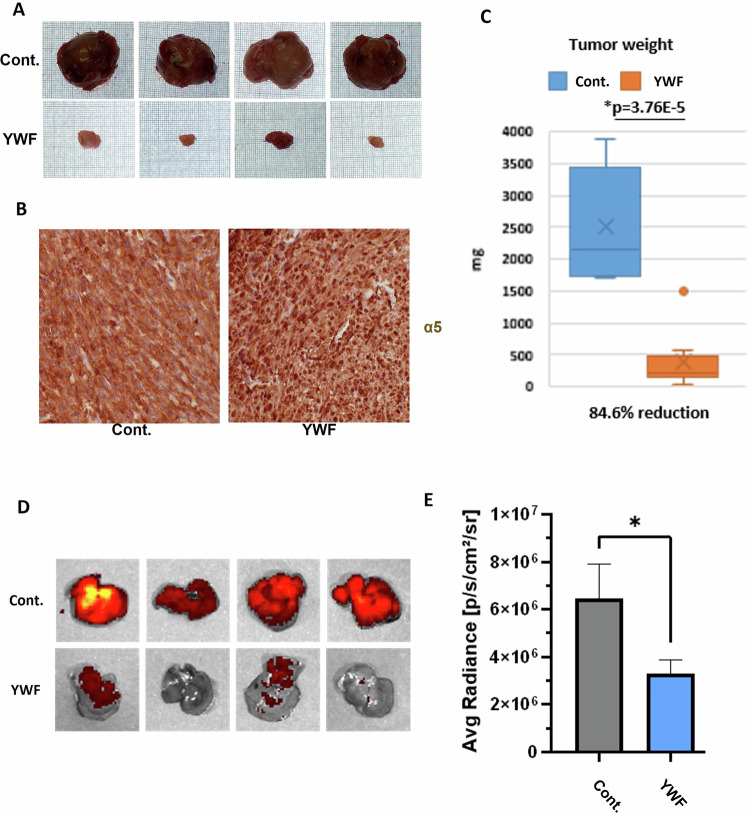
Nuclear proteasome sequestration using YWF is effective against tumors of different origins, as well as metastasis. **A** The carcinogen 3-MCA was injected once into the thigh of immune-competent mice, resulting in the formation of a sarcoma, at which point the animals were treated as indicated (see also under “Materials and methods”). Mice were sacrificed, tumors were harvested, and tissue sections were fixed in formaldehyde and embedded in paraffin. Presented are gross samples from the time of animal sacrifice, photographed for scale on a graph paper. **B** Immunohistochemistry of the proteasome in histopathological sections from the sarcomas generated as described under **A**. **C** Sarcomas were weighed at the time of sacrifice, and tumor weights were plotted, demonstrating the therapeutic effect of YWF. **D** To generate allograft breast tumors, 4T1 murine breast carcinoma cells expressing a fluorescent marker were inoculated in the mammary glands of female immune-competent mice. Following the formation of tumors, the animals were treated as indicated (see also under “Materials and methods”). Mice were sacrificed, and their livers were isolated and visualized to assess the intensity of fluorescence using IVIS. The fluorescence intensity correlates with the extent of metastases from the primary allograft in the mammary gland. **E** Analysis of mCherry intensity as measured by IVIS, as described under **D**.

After demonstrating the effect of YWF-induced proteasome sequestration to the nucleus in different types of epithelial tumor models, we next aimed to broaden the repertoire of tumors also to an aggressive mesenchymal tumor. We therefore used a carcinogen-induced sarcoma model, induced by a single subcutaneous injection of 3-Methylcholanthrene (3-MCA) to the animals’ thigh. YWF treatment, initiated at the point where the sarcomas were palpable, resulted in smaller tumors and nuclear proteasome sequestration (Fig. 8A–C).

While many tumors are diagnosed and classified based on the sampling of the primary mass, metastases are the leading cause for cancer’s lethality [48]. At the same time, metastasis is a complex process, and for cancer cells to migrate and colonize distant tissues they must overcome several hurdles, including the metabolic challenges imposed by the microenvironment in both their origin and target sites [49]. We therefore tested the effect of inhibiting proteasome recruitment also on metastases, using the 4T1 triple-negative breast cancer model [50]. These aggressive and rapidly metastasizing breast carcinoma cells (marked by mCherry) were injected into the mammary gland of WT female mice, which were then monitored – at this stage without any treatment. Notably, although the primary tumor in this model is the result of exogenously injected cells, the metastatic phase shares common features with human metastatic breast cancer [51]. Using this model, we found that in addition to their inhibitory effect on the growth of primary tumors (Figs. 5–7), also metastasis can be inhibited by the triad: the extent of liver metastases from breast carcinoma was significantly lower following YWF treatment (Fig. 8D and E). These findings probably reflect the challenging metabolic stress experienced by metastasizing cells, as well as other essential stress-coping, invasion, and growth mechanisms that are dependent on adequate proteolysis which is interrupted by proteasomal nuclear sequestration.

In summary, our findings unravel a key role for proteasome dynamics as an essential stress-coping mechanism in solid tumors, and a potential target for the development of therapeutic modalities.

## Discussion

In our previous study, we demonstrated that starvation to amino acid results in translocation of the proteasome from its large storage in the nucleus to the cytosol, in order to increase proteolytic activity in the larger cell compartment. We found that the translocation is mediated by the mTOR pathway, sensing specifically the lack of the three aromatic amino acids, YWF. The supplementation of the triad alone activates mTOR which results in sequestration of the proteasome in the nucleus, and induction of cell death, resulting from the lack of all other 17 amino acids, which otherwise would have originated from cytosolic protein degradation [32].

In the current work, we have unraveled that this mode of regulation of proteasome cellular compartmentation is operational not only in cultured cells, but also in the intact animal (Figs. 5–8). Furthermore, we found that both proteasome recruitment and its inhibition by YWF are not limited only to amino acid starvation, but occur also under other stress conditions, such as hypoxia (Fig. 1). Unlike cultured cells, it is impossible to deprive animals entirely from amino acids, as severe starvation to dietary proteins will stimulate degradation of endogenous proteins, such as muscle proteins [52, 53]. This led us to examine the effect of the aromatic amino acid triad on tumors, which are inherently stressed, and therefore their proteasome is largely localized to the cytosol (Fig. 4). We hypothesized that introduction of excess of YWF will transduce a signal of false satiety also in the stressed tumor cells, which will result in sequestration of their proteasome in the nucleus, subsequent cell death, and tumor shrinkage. This was indeed the case, in both xenografts implanted in immune compromised mice (Figs. 4 and 5), and in endogenous tumor models in immune competent mice (Figs. 6–8).

Transcriptomic and proteomic analyses revealed multiple pathways that are modulated by introduction of YWF (Figs. 2 and 3). While some of them represent pathways related to cell fate, other are not. Similarly, certain post-translational modifications point to the effect of YWF on the same functions, but others do not. Further studies are required in order to shed light on the role of these altered pathways, and how they are linked to the mechanism of proteasomal translocation, and its cytotoxic effect(s).

The UPS itself is already targeted by approved treatments for malignancies, both in the form of proteasome inhibitors that directly target the system [54], as well as immune modulatory drugs (IMiDs) such as lenalidomide. These drugs target driver proteins in malignant cells for ubiquitination and subsequent degradation by the proteasome [55–57], and therefore rely on the adequate activity of the UPS. The incidental discovery of thalidomide as a molecular glue opened a new field, and different strategies that target the UPS for therapeutic uses are currently under extensive research and development [58–60].

Notably, it was previously shown that adequate protein quality control is essential physiologically [61], and that the mTOR pathway plays key roles at the organ and organism level [62–64]. Our findings now reveal another aspect of its activity, as well as the potential benefit of its manipulation. The identification of proteasome dynamics as a potential therapeutic target in cancer further underscores the central role the UPS plays in various cellular functions, as well as its requirement for cancer cell survival, proliferation, and tumor growth. It also presents a unique therapeutic approach, as it is not a chemotherapy or any other modality currently used for treatment. Given that drugs of different natures are often combined for the treatment of a certain malignancy, including the case of proteasome inhibitors and IMiDs in multiple myeloma [65], clinical use of the modulation of proteasome dynamics may turn out as beneficial when used in combination with drugs from other families. One way or another, the expansion of the anti-cancer therapeutic armamentarium is clearly required, as resistance to existing drugs is constantly developing [66].

This study also identified SESN3 as a potential oncogene, given its role in mTOR-mediated proteasome translocation in response to stress. We found that following knockdown of SESN3, the tumors originating from inoculated human cells were dramatically smaller ( > 90% reduction in size) relative to their wild type counterparts. This finding calls for further research into SESN3’s mechanism in sensing YWF (is it a direct binder of this triad, and why all three are needed?), and its role in oncogenesis. It also points to its potential use as a druggable target.

## Materials and methods

### Immunofluorescence microscopy

Cells were seeded on glass cover slips for 36 h. Following the indicated treatments, they were fixed with 4% PFA for 15 min, washed with phosphate-buffered saline (PBS) and incubated in PBS containing 10% goat serum for 1 h at room temperature, followed by 2 h incubation with the indicated primary antibody. Following extensive wash with PBS, the fixed cells were incubated with the relevant secondary antibody for 1 h, washed and mounted. Images were acquired using Zeiss LSM 700 confocal microscope (Zeiss, Oberkochen, Germany).

### Sample preparation for protein mass spectrometry

Three replicates of 2–3 mg of cell extract protein in 8 M Urea and 100 mM ammonium bicarbonate, were incubated with DTT (2.8 mM; 30 min at 60 °C), modified with iodoacetamide (8.8 mM; 30 min at room temperature in the dark), and digested (overnight at 37^0^C) with modified trypsin (Promega; 1:50 enzyme-to-substrate ratio) in 2 M urea and 25 mM ammonium bicarbonate. Additional second trypsinization was carried out for 4 h. The tryptic peptides were desalted using Sep-Pak C18 (Waters) and dried. Ten micrograms of protein were used for proteome analysis as described under Mass spectrometry. Enrichment for ubiquitinated peptides was done as previously described [67, 68], using bead-conjugated anti-K-GG antibody (PTMScan Ubiquitin Remnant Motif, Cell Signaling).

### Proteins mass spectrometry

Tryptic peptides were analyzed by LC–MS/MS using a Q Exactive plus mass spectrometer (Thermo Fisher Scientific) fitted with a capillary HPLC (easy nLC 1000, Thermo). The peptides were loaded onto a C18 trap column (0.3 × 5 mm, LC-Packings) connected online to a home-made capillary column (20 cm, internal diameter 75 microns) packed with Reprosil C18-Aqua (Dr. Maisch GmbH, Germany) in solvent A (0.1% formic acid in water). The peptides mixture was resolved with a 5–28% linear gradient of solvent B (95% acetonitrile with 0.1% formic acid in water) for 180 min followed by a 5 min gradient of 28–95% and 25 min at 95% acetonitrile with 0.1% formic acid at a flow rate of 0.15 μl/min. Mass spectrometry was performed in a positive mode (*m*/z 350–1800, resolution 70,000) using repetitively full MS scan followed by collision-induced dissociation (HCD at 35 normalized collision energy) of the 10 most dominant ions (>1 charges) selected from the first MS scan. A dynamic exclusion list was enabled with exclusion duration of 20 sec.

### Proteomics data analysis

The mass spectrometry raw data were analyzed by the MaxQuant software (version 1.4.1.2, http://www.maxquant.org) for peak picking and quantification. This was followed by identification of the proteins using the Andromeda engine, searching against the human UniProt database with mass tolerance of 20 ppm for the precursor masses and for the fragment ions. Met oxidation, N-terminal acetylation, N-ethylmaleimide and carbamidomethyl on Cys, GlyGly on Lys, and phosphorylation on Ser, Thr and Tyr residues, were set as variable post-translational modifications. Minimal peptide length was set to six amino acids and a maximum of two mis-cleavages was allowed. Peptide and protein levels false discovery rates (FDRs) were filtered to 1% using the target-decoy strategy. Protein tables were filtered to eliminate identifications from the reverse database and from common contaminants. The MaxQuant software was used for label-free semi-quantitative analysis [based on extracted ion currents (XICs) of peptides], enabling quantification from each LC/MS run for each peptide identified in any of the experiments. In samples that were SILAC-labeled, quantification was also carried out using the MaxQuant software. Data merging and statistical tests were done by the Perseus 1.4 software.

### Mass spectrometric analysis of proteasome sub-complexes’ composition

Analysis of proteasome assembly and sub-complex composition was performed using the MCP20 antibody against the α6 proteasome subunit, as previously described using the MCP21 antibody against α2 [69]. Briefly, following the indicated treatments, cell lysates were immunoprecipitated using the α6 antibody, and the precipitates were subjected to proteomic mass spectrometry. Intensities of the different proteasomal subunits were normalized according to the median of intensities measured for all 20S subunits. Ratios were calculated for each condition relative to untreated cells (Control). The color scale represents Log2 fold-change.

### RNA extraction and quality control

Total RNA was extracted from four replicates of cell pellet samples using the QIAcube Connect (Qiagen) with the RNeasy mini kit (Qiagen, cat no. 74106) according to the manufacturer’s protocol. The quality of the RNA was evaluated using the TapeStation 4200 (Agilent) with the RNA kit (Agilent, cat no. 5067–5576). The RINe values of all the samples were 10, indicating best high-quality RNA.

### RNA library preparation and sequencing

RNA-seq libraries were constructed using NEBNext Ultra II Directional RNA Library Prep Kit for Illumina (NEB, cat no. E7760), according to the manufacturer’s protocol. 800 ng total RNA was used as the starting material. mRNA pull-down was performed using the NEBNext® Poly(A) mRNA Magnetic Isolation Module (NEB, cat no. E7490). RNA-seq library QC was performed by measuring library concentration using Qubit (Invitrogen) with the Equalbit RNA HS Assay Kit (Vazyme, cat no. EQ211) and size determination using the TapeStation 4200 (Agilent) with the D1000 kit (Agilent, cat no. 5067–5582). All libraries were mixed into a single tube with equal molarity.

The RNA-seq data was generated on Illumina NextSeq2000, using P3 50 cycles (Read1–72; Index1–8; Index2–8) (Illumina, cat no. 20046810).

### Bioinformatics analysis

Quality control was assessed using FastQC (v0.12.1) and MultiQC (v1.14). Reads were trimmed for adapters, minimum quality Phred score of 20 and minimum length of 35 bases using Trim Galore (v0.6.10) which applies Cutadapt (v4.4). Single reads (72 bp) were aligned to Homo sapiens GRCh38 (https://ftp.ensembl.org/pub/release-109/fasta/homo_sapiens/dna/Homo_sapiens.GRCh38.dna.primary_assembly.fa.gz) and annotation file (https://ftp.ensembl.org/pub/release-109/gtf/homo_sapiens/Homo_sapiens.GRCh38.109.gtf.gz) using STAR (v2.7.10b) with mismatch ratio allowed < 0.2, the minimum and maximum intron sizes were set to 21 and 1,000,000, respectively. The number of reads per gene was counted using STAR “--quantMode GeneCounts” (v2.7.10b) with ‘reverse’ mode. Normalization and differential expression analyses were conducted using DESeq2 R package (v1.36.0). The threshold for significantly differentially expressed genes is determined by two factors: adjusted p-value ≤ 0.05 and the ‘base-mean independent filtering’ threshold, which is calculated by the DESeq2 algorithm for each comparison. FDR was calculated using the default approach of DESeq2, Benjamini-Hochberg.

### Cell survival assay

Cells were seeded in a 96-well plate at a density of 15,000 cells/well. ~36 h later, cells were treated as described and were visualized live, using high-throughput fluorescence microscopy (IXM-C, Molecular Devices) under a controlled environment (21% O_2_, 5% CO_2_, 37 °C). Hoechst 33342 was used to stain nuclei of all cells, and SYTOX^TM^ (Thermo) was used to stain dead cells. Data analysis was performed using the Live/Dead module of the MetaXpress software (Molecular Devices).

### Statistics, reagents, and animal models

No sample size calculations and blinding were performed. The inclusion criterion was the development of a tumor. Animals were randomized to the different experimental groups. No samples were excluded from analysis. Two-tailed Student’s t test was used. In box-plots, x represents the average, while the box and bars represent quartiles.

### Xenografts

MDA­MB­231 (ATCC® HTB­26™) or HeLa (ATCC® CCL-2™) cells, both validated by STR profiling and tested for mycoplasma, were dissociated with trypsin, washed with PBS, and brought to a concentration of 70 × 10^6^ cells/ml. Cell suspension (7 × 10^6^/0.1 ml) was inoculated subcutaneously at the flanks of 12 weeks old NOD.Cg-Prkdc^scid^Il2rg^tm1Wjl^/SzJ (NSG) mice, JAX stock #005557. Following formation of a palpable mass (MDA-MB-231 *n* = 16; HeLa *n* = 8). For subcutaneous administration, 500 μl of either saline, saline supplemented with 25 mM/each of YWF, or saline supplemented with 25 mM/each of QLR, was injected 3 times a week, in adjacent to the tumor. For oral treatment, YWF were dissolved in the drinking water at a concentration of 6 mM each. After the largest tumor in each experiment has reached the maximal size allowed by the guidelines for animal care, all mice were sacrificed and xenografts were resected, weighed, and fixed in formalin. Paraffin-embedded sections were stained using standard immunohistochemistry protocol as described previously [70]. Apoptotic cells were detected using terminal deoxynucleotidyl transferase dUTP nick end labeling (TUNEL) according to the manufacturer’s protocol, and via immunofluorescence against the apoptotic marker cleaved-Caspase3. Volumetric monitoring of tumors was carried out using a caliper twice a week. All animal experiments were carried out under the supervision of the accredited Animal Care Committee of the Technion.

### Spontaneous tumor models

For the colorectal tumor model, APC^fl/fl^ mice were kindly provided by E. Fearon (University of Michigan, Ann Arbor, MI). CDX2-CreER^T2^ mice were purchased from the Jackson laboratory. All transgenic mice were on C57BL/6J background. The mice were crossed to generate APC^fl/fl^ CDX2-CreER^T2^ mice. To get discrete colonic tumors we calibrated a model based on a low dose tamoxifen. Tamoxifen (Sigma) was diluted in corn oil (Sigma) and injected intraperitoneally in a single dose of 20 mg/kg (*n* = 5). For YWF treatment, YWF at a concentration of 6 mM was administered in the drinking water starting 10 Days after tamoxifen injection, and mice were sacrificed 7 weeks later. Colonic tumors exceeding 0.5 mm were measured using a digital caliper. Tumors in the cecum are hard to measure due to the irregularity of the surrounding cecal tissue. Thus, cecum mass was measured and compared to APC^fl/fl^ non-induced mice, to estimate the change in tumoral mass in the cecum. Tissue was fixed in 4% formaldehyde and FFPE blocks were prepared for histologic analysis.

Bladder carcinoma was induced adding the carcinogen N-Butyl-N-(4-hydroxybutyl)nitrosamine (BBN) to the drinking water as previously described [47] (*n* = 14). Sarcomas were induced by a single subcutaneous injection of 3-Methylcholanthrene (3-MCA), as previously described [71] (*n* = 14).

Carcinogens used:

**Table:** 

N-Butyl-N-(4-hydroxybutyl)nitrosamine	Santa Cruz	0.05% (W/V)
3-Methylcholanthrene	Santa Cruz	200 µg/mouse

### Metastatic triple-negative breast cancer model

Nine weeks old BALB/c female mice (Envigo, Jerusalem, Israel) were injected subcutaneously with 4 × 10^5^ 4T1 mCherry-expressing cells in 50 μl PBS to the lower left mammary fat pad (n = 9). Mouse weights were monitored and tumor dimensions were measured by a caliper 3 times a week. Tumor volume was defined as (length) × (width)^2^/2.

Metastases detection: following mice sacrificing, the livers were harvested and imaged using IVIS Spectrum CT Pre-Clinical In Vivo Imaging System (PerkinElmer, MA, USA) at ex/em of 570/620 nm, binning of 2, f-stop of 2, and a 10 s exposure time to detect mCherry metastases. Quantitative data from the images were obtained using ROI tool in Living Image software. Non-inoculated control mice were used for the analysis, and the average radiance of their unaffected livers was used as a baseline.

### Cell culture treatments

For starvation, cells were washed twice with PBS, followed by incubation with a medium lacking all amino acids.

Starvation medium: Dulbecco’s Modified Eagle Medium (DMEM) with a formulation as follows:

**Table:** 

*Components*	*Concentration (mg/L)*
**Inorganic salts**	
CaCl_2_·2H_2_O	264.92
Ferric nitrate (Fe(NO_3_)3-9H_2_O)	0.10
Potassium chloride (KCl)	400.00
MgSO4·7H_2_O	200.00
Sodium chloride (NaCl)	6400.00
Sodium bicarbonate (NaHCO_3_)	3700.00
Sodium phosphate (NaH_2_PO_4_-H_2_O)	125.00
**Other components**	
D-Glucose	4500.00
Phenol Red	15.00
**Vitamins**	
D-Calcium pantothenate	4.00
Choline chloride	4.00
Folic acid	4.00
i-Inositol	7.20
Niacinamide	4.00
Pyridoxine HCl	4.00
Riboflavin	0.40
Thiamine HCl	4.00

For treatment with LMB, Ivermectin, Phenformin, Ionomycin, and 2-DG, cells were incubated for 8 h in a medium supplemented with the appropriate reagent, using concentrations as follow:

**Table:** 

Reagents	Supplier	Concentration
Leptomycin B	Sigma	2.5 ng/ml
Ivermectin	Sigma	20 µM
Phenformin	Sigma	0.5 µM
Ionomycin	Sigma	1 µM
2-DG	Sigma	2 mM

**Table:** 

Antibodies	Supplier	Cat#
β4	Santa Cruz Bio Technology	sc-515066
α5	Cell Signaling Technologies	2457
α6	Enzo	BML-PW8100
PROX1	Abcam	ab-199359
Cleaved-caspase3	Cell Signaling Technologies	9661
Lamin A/C	Santa Cruz Bio Technology	Sc-7292

## Supplementary information

Original data files

Figure S1

Figure S2

Figure S3

Figure S4

Figure S5

Figure S6

Supplementary Figure Legends

Supplemental Table

## Data Availability

The datasets used in the current study are available on the MassIVE repository (ftp://massive.ucsd.edu/v08/MSV000095640/).
