# Supplementary figures and images for: Inhibition of nucleo-cytoplasmic proteasome translocation by the aromatic amino acids or silencing Sestrin3—their sensing mediator—is tumor suppressive
Source: Cell Death Differ. 2024 Sep 12;31(10):1242–54. doi: 10.1038/s41418-024-01370-x (PMC11445514; doi:10.1038/s41418-024-01370-x)

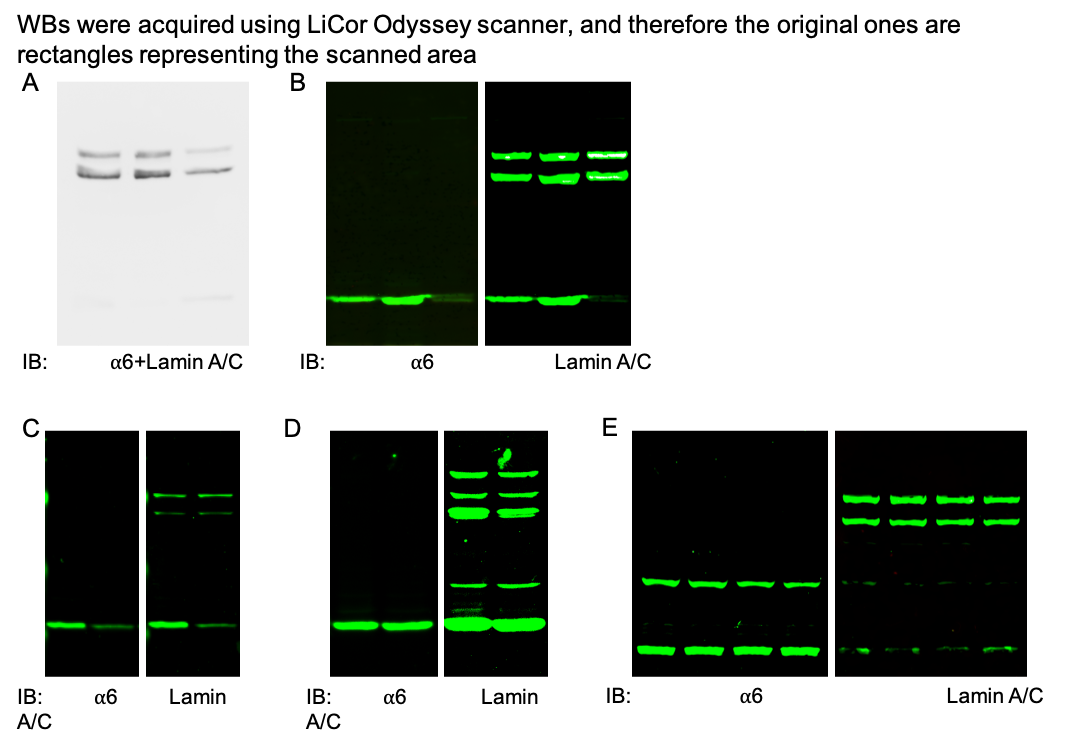

Supplement: Supplementary file 1 — Original data files [file 41418_2024_1370_MOESM1_ESM.png]

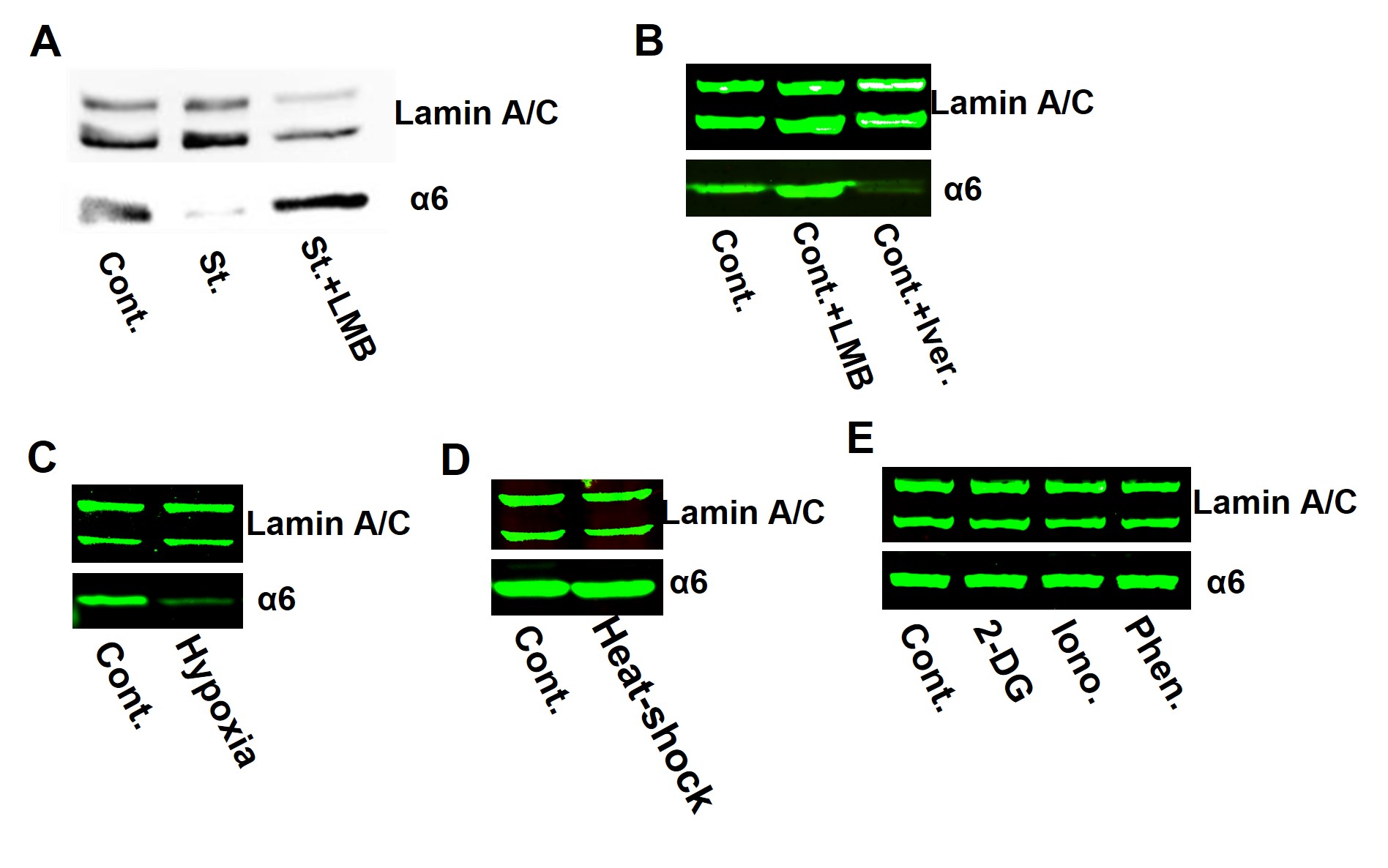

Supplement: Supplementary file 2 — Figure S1 [file 41418_2024_1370_MOESM2_ESM.jpg]

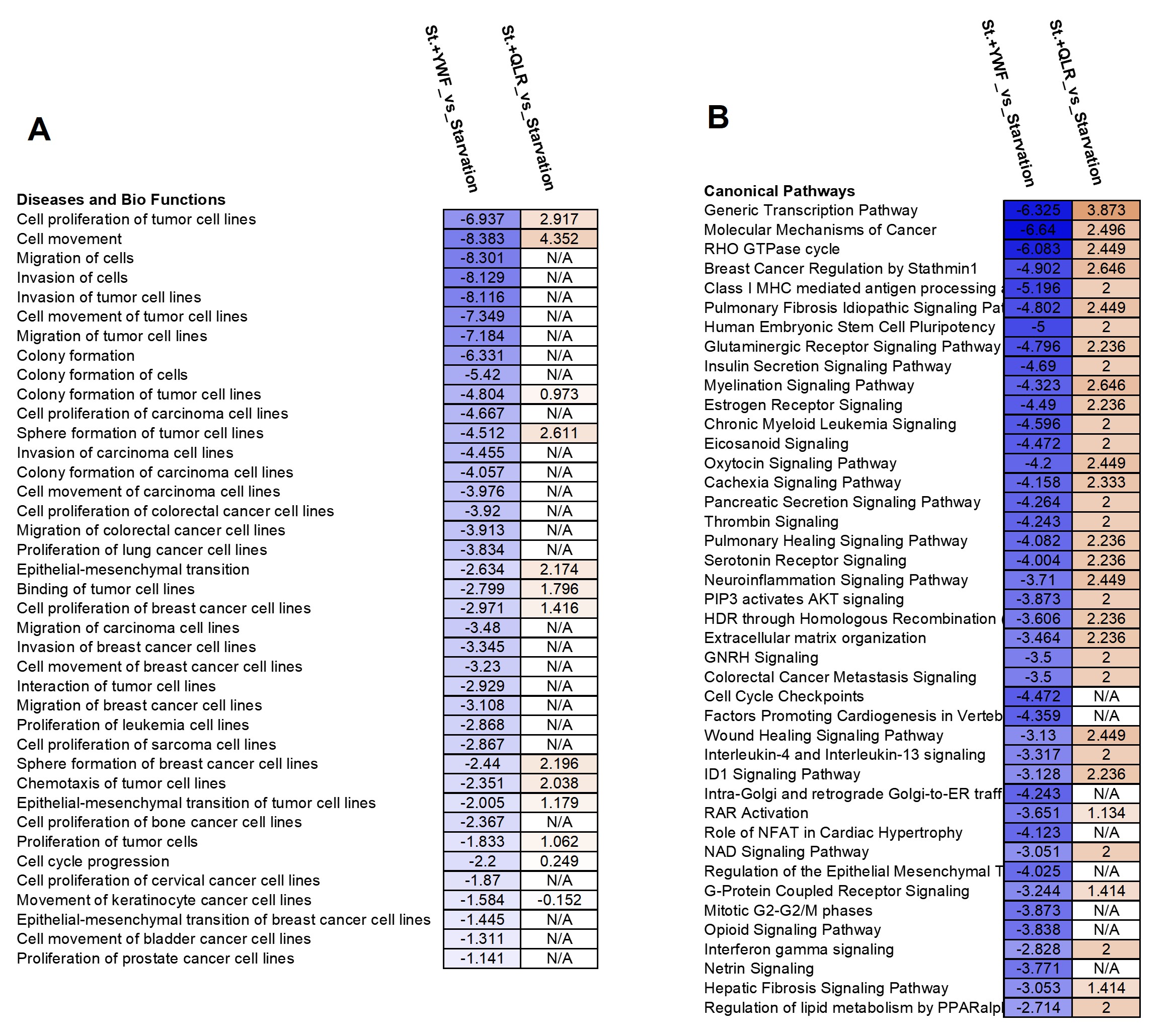

Supplement: Supplementary file 3 — Figure S2 [file 41418_2024_1370_MOESM3_ESM.jpg]

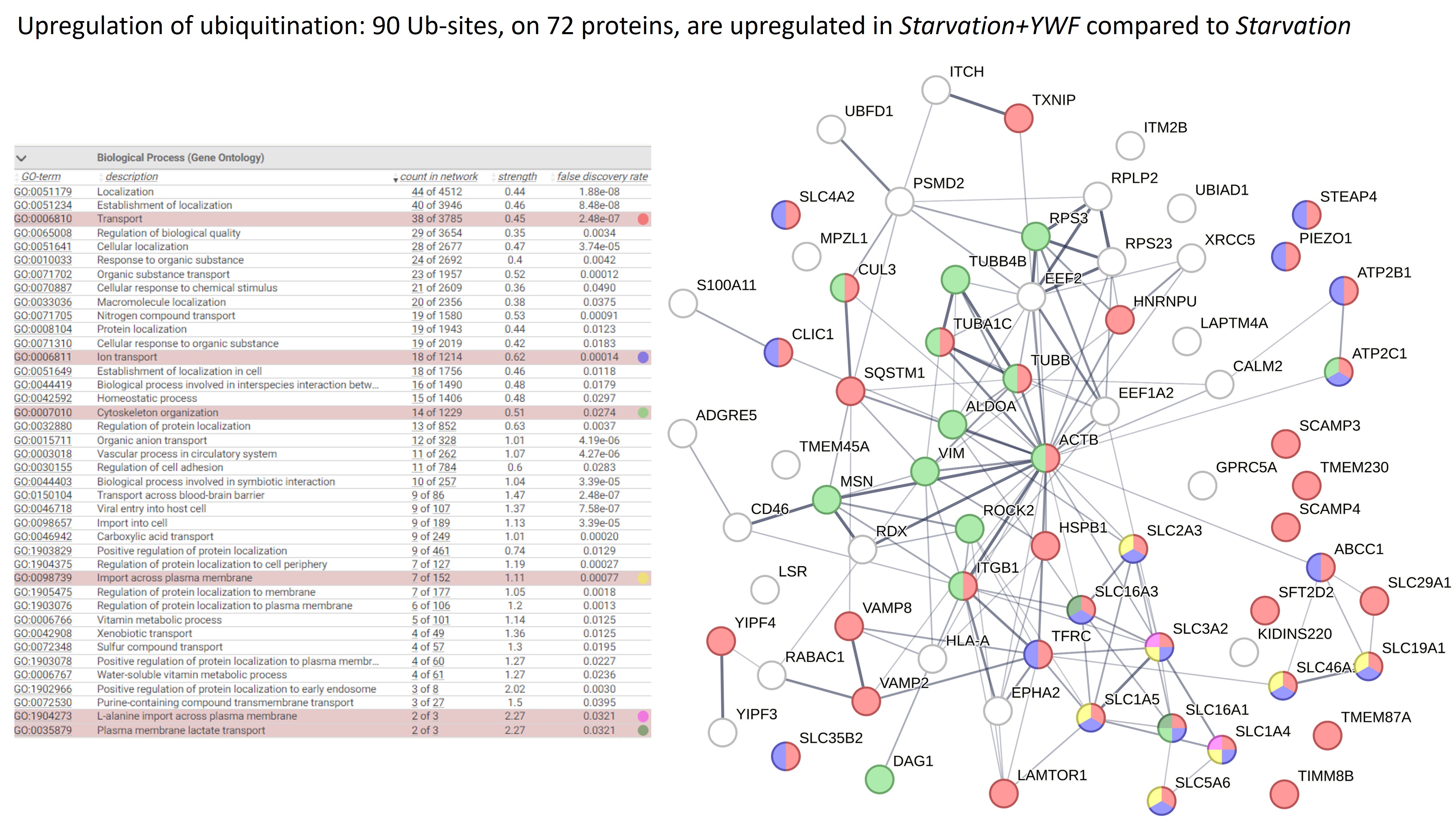

Supplement: Supplementary file 4 — Figure S3 [file 41418_2024_1370_MOESM4_ESM.jpg]

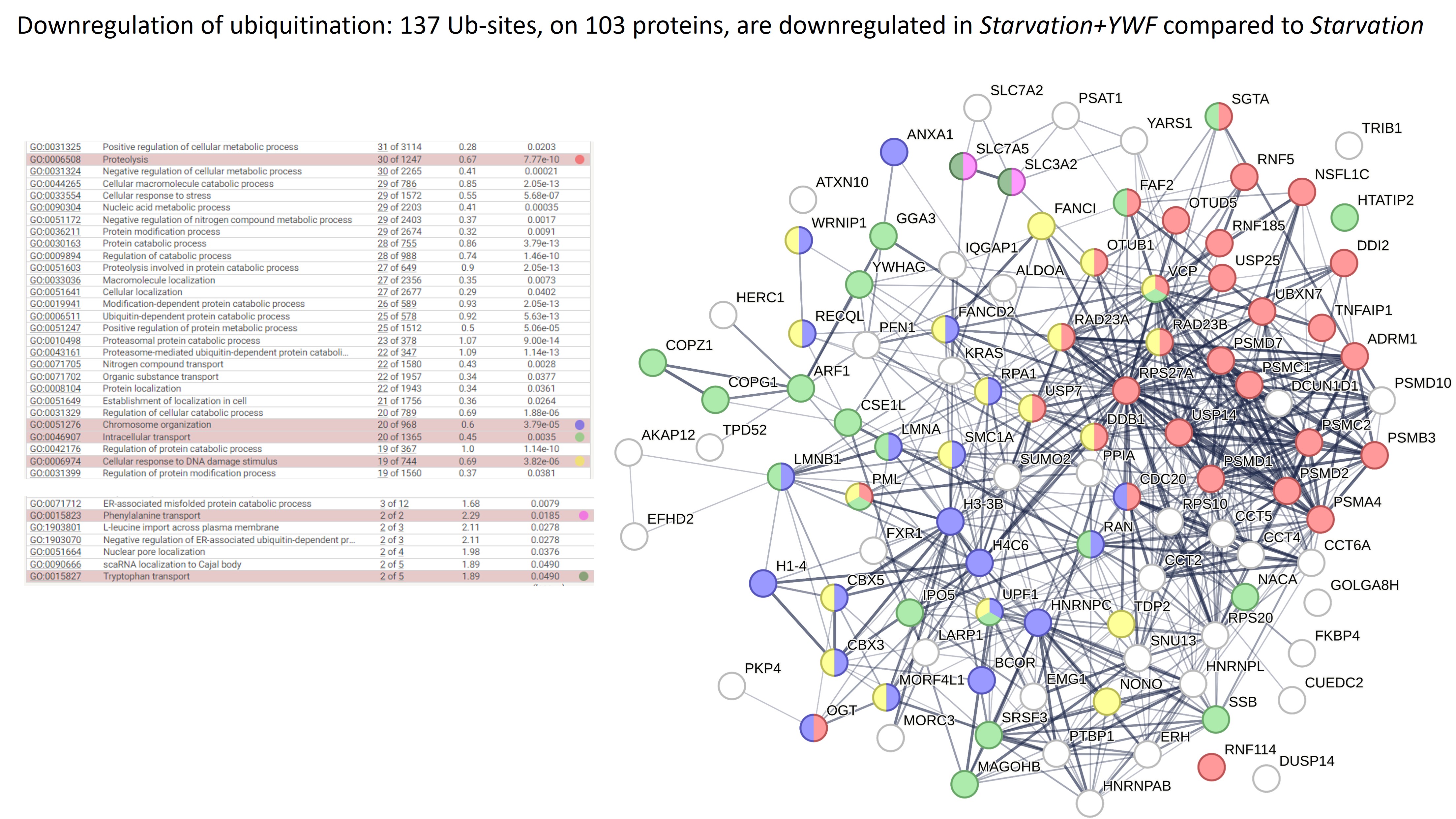

Supplement: Supplementary file 5 — Figure S4 [file 41418_2024_1370_MOESM5_ESM.jpg]

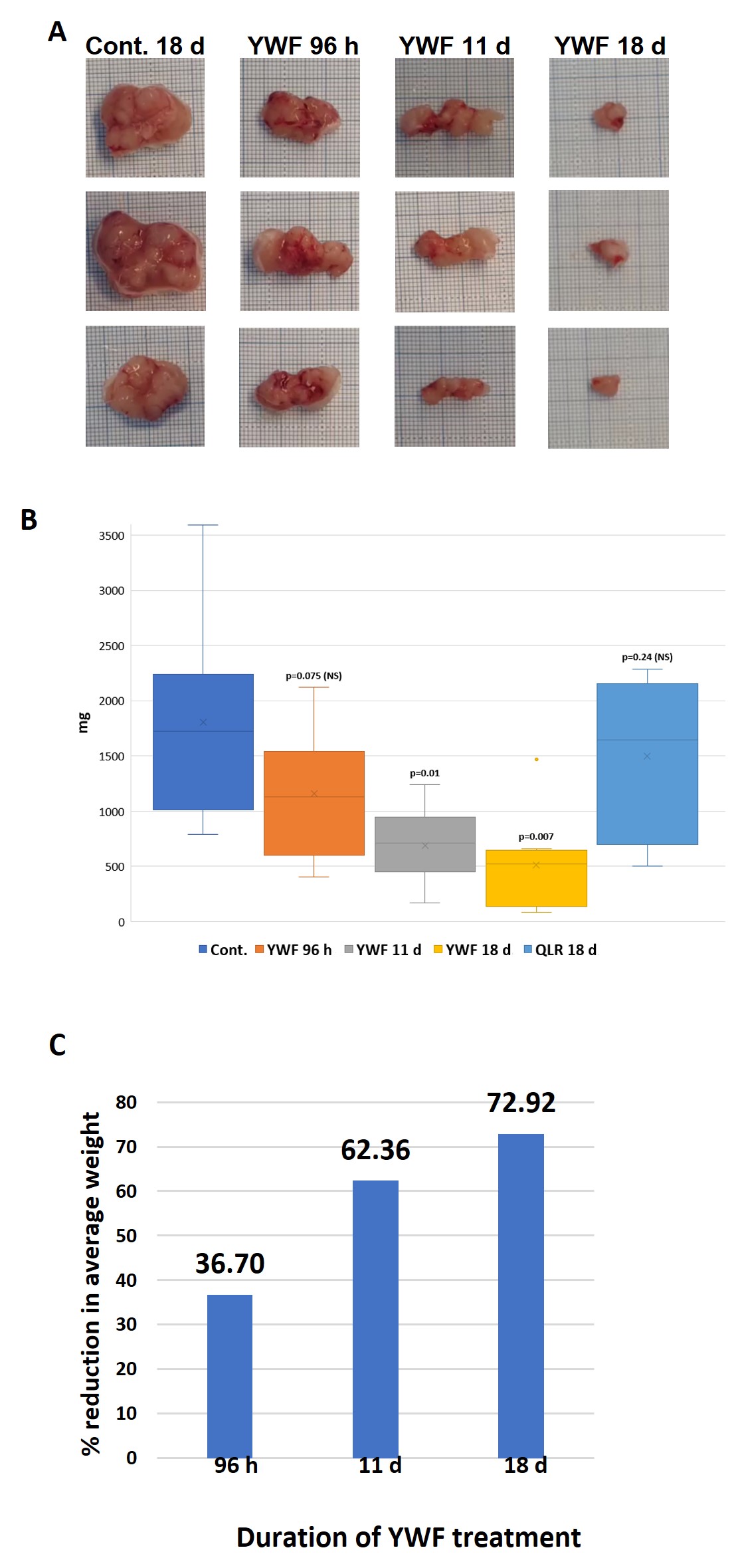

Supplement: Supplementary file 6 — Figure S5 [file 41418_2024_1370_MOESM6_ESM.jpg]

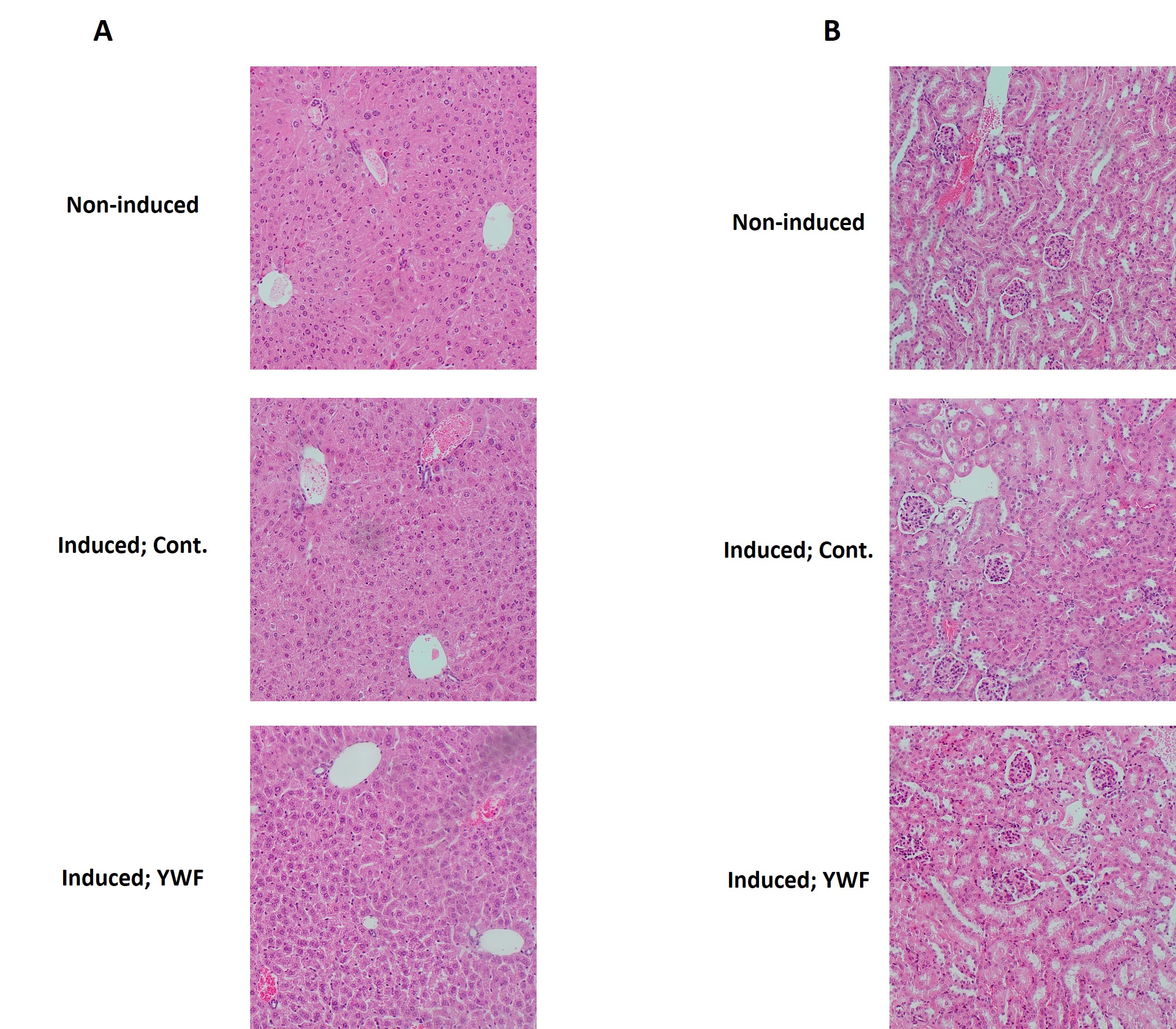

Supplement: Supplementary file 7 — Figure S6 [file 41418_2024_1370_MOESM7_ESM.jpg]
