# Supplementary material for: Inhibition of nucleo-cytoplasmic proteasome translocation by the aromatic amino acids or silencing Sestrin3—their sensing mediator—is tumor suppressive
Source: Cell Death Differ. 2024 Sep 12;31(10):1242–54. doi: 10.1038/s41418-024-01370-x (PMC11445514; doi:10.1038/s41418-024-01370-x)
Supplement: Supplementary file 8 — Supplementary Figure Legends [file 41418_2024_1370_MOESM8_ESM.docx]

Supplementary Figure Legends

**Figure S1, related to main Figure 1:**

Cells were treated as indicated, nuclear fractions were purified from cell lysates, resolved via SDS-PAGE, and blotted for the proteasome and the nuclear marker Lamin A/C.

**Figure S2, related to main Figure 2:**

HeLa cells were incubated in either a complete medium, starvation medium, starvation medium supplemented with Tyr, Trp, and Phe (St.+YWF), or starvation medium supplemented with Gln, Leu, and Arg (St.+QLR). Cells were harvested, and RNA was extracted and sequenced. Presented are different comparisons according to the Ingenuity Pathway Analysis, representing changes in known functions following the addition of either YWF (St.+YWF_vs_Starvation) or QLR (St.+QLR_vs_Starvation). The altered functions are clustered according to cancer cell proliferative and migratory traits (A), and broad general cellular activities (B).

**Figure S3, related to main Figure 3:**

A list and STRING network of proteins, the ubiquitination of which is upregulated following treatment with YWF.

**Figure S4, related to main Figure 3:**

A list and STRING network of proteins, the ubiquitination of which is downregulated following treatment with YWF.

**Figure S5, related to main Figure 5:**

A. Tumors originating from HeLa cells were treated with YWF for the indicated times prior to sacrificing the mice. Tumors were photographed for scale on a graph paper.
B. Tumors were generated and treated as in A. Plotted are tumor weights at the time of mouse sacrificing.
C. Tumors were generated and treated as in A. Presented is the reduction in tumor weights relative to Control.

**Figure S6, related to main Figure 7:**

Gastrointestinal tumors were induced in immune-competent mice via the loss of the tumor suppressor gene adenomatous polyposis coli (APC; see also Figure 7 and under *Materials and Methods*). The animals were treated as indicated, and at the time of animal sacrifice and tumor harvesting, the liver (A), and kidney (B) were removed as well. Tissue sections were fixed in formaldehyde and embedded in paraffin, and histopathological slides were stained using H&E.
